# Low-intensity pulsed ultrasound: Nonunions

**DOI:** 10.4103/0019-5413.50848

**Published:** 2009

**Authors:** Bernadetta G Dijkman, Sheila Sprague, Mohit Bhandari

**Affiliations:** Division of Orthopaedic Surgery, Department of Surgery and Department of Clinical Epidemiology & Biostatistics. McMaster University, 293 Wellington Street North, Suite 110, Hamilton, Ontario L8L 2X2

**Keywords:** Fracture healing, low-intensity pulsed ultrasound (LIPUS), nonunion

## Abstract

Nonunions occur in 5–10% of fractures and are characterized by the failure to heal without further intervention. Low intensity pulsed ultrasound therapy has been developed as an alternative to surgery in the treatment of nonunions. We describe a systematic review on trials of low-intensity pulsed ultrasound therapy for healing of nonunions. We searched the electronic databases Medline and the Cochrane library for articles on ultrasound and healing of nonunions published up to 2008. Trials selected for the review met the following criteria: treatment of at least one intervention group with low intensity pulsed ultrasound; inclusion of patients (humans) with one or more nonunions (defined as “established” or as a failure to heal for a minimum of eight months after initial injury); and assessment of healing and time to healing, as determined radiographically. The following data were abstracted from the included studies: sample size, ultrasound treatment characteristics, nonunion location, healing rate, time to fracture healing, fracture age, and demographic information. We found 79 potentially eligible publications, of which 14 met our inclusion criteria. Of these, eight studies were used for data abstraction. Healing rates averaged 87%, (range 65.6%-100%) among eight trials. Mean time to healing was 146.5 days, (range 56-219 days). There is evidence from trials that low-intensity pulsed ultrasound may be an effective treatment for healing of nonunions. More homogeneous and larger controlled series are needed to further investigate its efficacy.

## INTRODUCTION

Nonunions occur in 5-10% of fractures and are characterized by the failure to heal without further intervention.^1^,^2^ To avoid the risks of surgery, several alternative treatments have been developed, including electrical stimulation, extracorporeal shock wave therapy, and low-intensity pulsed ultrasound (LIPUS). LIPUS has been found to have a beneficial effect on fracture healing by increasing the quantity and strength of bony callus and by several other biological and molecular mechanisms.^3^–^5^ Also, it is a safe and noninvasive treatment, since it uses mechanical energy of a low intensity, thereby not causing tissue damage.^6^,^7^ Although many randomized controlled trials (RCTs) have been conducted investigating the effect of LIPUS on fresh fractures,^8^–^10^ far less evidence is available on its efficacy on nonunions. We describe a systematic review of trials on LIPUS therapy used for healing of nonunions, to evaluate the current evidence on success rates of LIPUS therapy for nonunions. Also, we provide background information on mechanisms of LIPUS and potential factors influencing its efficacy.

## MATERIALS AND METHODS

### Search strategy

We searched the electronic databases of MEDLINE and the Cochrane library for articles on ultrasound and healing of nonunions published up to 2008. The search strategy consisted of the following terms: “fracture healing,” “bone graft,” “bone remodeling,” “nonunion,” “ununited,” and “ultras*,” in which the wild term “*” was used to allow all terms that start with the preceding letters, such as “ultrasound” and “ultrasonography.” We searched for all types of study designs (RCTs, cohort studies, case series, and case reports), and systematic reviews and meta-analyses. In addition, we searched in the bibliographies of all retrieved articles to find other relevant articles.

### Eligibility criteria

For each potentially eligible publication, each of the following inclusion criteria had to be met: treatment of at least one intervention group with LIPUS; inclusion of patients (humans) with one or more nonunions, as defined below; and assessment of healing and time to healing, as determined radiographically. There is no agreement among surgeons on the definition of a nonunion, ranging from 2 to 12 months after initial injury.^11^ To make sure the studies had included true nonunions only, we chose a rather conservative cutoff point for nonunion definitions. We included studies in which a nonunion was either called “established” (a minimum of nine months after initial injury and no signs of progression in the last three months),^12^ or defined as a fracture that had failed to heal within a minimum of 8 months after initial injury. Articles in any other language than English and articles on case reports were excluded from data abstraction.

### Data abstraction

The following data were abstracted from the included studies: study design, sample size, LIPUS treatment characteristics, nonunion location, healing rate, time to fracture healing, fracture age, and demographic information.

Reviews that were found by our search strategy were all evaluated by us on relevant information and references.

## RESULTS

### Study identification

We found 79 potentially eligible publications, of which 14 met our inclusion criteria and were reviewed by us. Of the 65 excluded articles, 43 did not use LIPUS as an intervention, 7 studies were excluded because they did not include nonunions, 10 were reviews, and 5 were animal or cell studies. Of the 14 included studies, 6 were excluded from the data abstraction.

Among the six studies excluded from the data abstraction, the study of Jones *et al*.^13^ used LIPUS as an adjunctive therapy to surgery, as a result of which healing could not be ascribed to the ultrasound treatment solely. The one RCT in our review also used LIPUS as an adjunctive therapy to surgery (vascularized pedicle bone graft), but was included since it was placebo-controlled, which enables us to attribute the healing effect to the LIPUS treatment. Another study^14^ was excluded because the authors did not define the fracture as a nonunion. Finally, four studies were excluded from data abstraction because they were case reports.

### Study characteristics and methodological quality

Table 1 presents the baseline characteristics and study design of the eight studies selected for review.^6^,^15^–^21^ One double-blinded RCT and seven case series were included, of which five were of a prospective, and two were of a retrospective design. The double-blinded RCT included 21 established nonunions of the scaphoid treated with vascularized pedicle bone graft in patients with a mean age of 26.7 years, all being male.

**Table 1:** Baseline characteristics and study design of the trials included in the review

Citation	Sample size (no. of nonunions)	Male: female ratio	Mean patient age (years)	Site of nonunion	Definition of nonunion	Study desig
Ricardo 2006^15^	21	21:0	26.7	Scaphoid	Established—not specified	Double-blinded RCT
Rutten 2007^6^	71	56:15	40	Tibia	Not united for ≥ 6 mo. post fracture, and ≥ 3 mo. no progression of healing	Prospective series
Nolte 2001^16^	29	17:12	47	Various	Not united for ≥ 6 mo. post fracture, and ≥ 3 mo. no progression of healing	Prospective series
Jingushi 2007^17^	21	-	40.4	Long bones	Additional operative treatment being indicated	Prospective series
Gebauer 2005^18^	67	41:26	46	Various	Not united for ≥ 8 mo. post fracture, and ≥ 3 mo. no progression of healing	Prospective series
Gebauer 2005a^19^	4	-	-	Tibia and femur	Not united for ≥ 8 mo. post fracture	Retrospective series
Mayr 2000^20^	366	-	-	Various	Not united for ≥ 9 mo. post fracture	Retrospective series
Pigozzi 2004^21^	15	12:3	35.5	Various	Not specified	Prospective series

In all the eight reviewed studies, the investigators applied a 20-minute daily LIPUS treatment to their treatment groups, with an ultrasound signal that was composed of a burst width of 200 μs containing 1.5 MHz sine waves, with a 1 kHz frequency and an intensity of 30 mW/cm^2^. In the one included double-blinded RCT, units were externally identical for the sham and active stimulation groups, and the sham units were adjusted to give no ultrasound signal output across the transducer.

### Healing rate

In the reviewed studies, the healing rate varied from 65.6%^17^ to 100%; the latter success rate found in three different studies [Table 2].^15^,^19^,^21^ The study with the highest level of evidence (the double-blinded RCT) reported a healing rate of 100% of 21 scaphoid nonunions with an average fracture age of 38.4 months. A healing rate of 86% was reported by the study with the largest sample size (366 nonunions).^20^ On average, the healing rate of nonunions after LIPUS treatment was 87% among the reviewed studies [Table 2].

**Table 2:** Summary of the results of the trials included in the review

	Outcome		Fracture characteristic	
		
Citation	Healing rate (%)	Mean time to healing (days)	Average fracture age (months)	Time since last surgery (months)
Ricardo 2006^15^	Active: 100	Active: 56	38.4	0
	Placebo: 100	Placebo: 94		
Rutten 2007^6^	73	184	8.6	6.5
Nolte 2001^16^	86	152	14.2	12
Jingushi 2007^17^	65.6	219	18.9*	11.5*
Gebauer 2005^18^	85	168	39	24.2
Gebauer 2005a^19^	100	Not reported	Not reported	Not reported
Mayr 2000^20^	86	152	25.2	Not reported
Pigozzi 2004^21^	100	94.7	11.2	Not reported
Average	87	146.5	22.2	10.8

* Based on the total sample of the trial, consisting of 51 delayed unions and 21 nonunions.

### Time to fracture healing

The time until union ranged from 56 to 219 days, with an average of 146.5 days in the eight included trials [Table 2]. The RCT reported a significant decrease in healing time of 38 days in the active LIPUS group compared with the placebo group (56±3.2 days compared with 94±4.8 days). Similar as to the healing rate, the average time to healing of the fracture approximates the time to healing found in the largest study (152 days).^20^

## DISCUSSION

### Fracture healing

Fracture healing is a highly complex process including three predominant stages, that is, the inflammatory phase, reparative phase, and remodeling phase. For this process, a large cell population (fibroblasts, macrophages, chondroblasts, osteoblasts, and osteoclasts) and the expression of relevant genes (controlling matrix production and organization, growth factors, and transcription factors) are needed at the right time and place for an undisturbed progress of fracture healing.^22^ Considering the complexity of fracture healing, it seems plausible that 5–-10% of fractures become a delayed union or nonunion.^1^

### Nonunion - Definition

Authors disagree in the definition of a nonunion with regard to the time from injury at which a nonunion is declared, ranging from 15 weeks^23^ to 12 months from injury.^24^ However, they all add to their definition that all fracture repair processes have stopped. U.S. Food and Drug Administration (FDA) states that “A nonunion is considered to be established when a minimum of nine months has elapsed since injury and the fracture site shows no visibly progressive signs of healing for minimum of three months.”^12^ In addition, consensus among authors exists on the definition of an established nonunion, as being one that does not heal without operation.^2^,^25^,^26^ Therefore, most studies on treatment of nonunion use a spontaneous healing rate of 0%, with which they compare the healing rate accomplished after the treatment under investigation. However, some studies have used an estimated spontaneous healing rate of 5–30% of nonunions for statistical analyses on the treatment effect.^6^,^27^,^28^

Although the cause of delayed union and nonunion is not known, several factors are associated with their occurrences.^22^,^29^ The major factors are those that characterize the injury, such as fracture location, comminution, vascular and soft tissue damage, bone loss, and infection. Also, several patient-related factors can be recognized, such as age, comorbidity, nutrition, smoking habits, and use of alcohol or drugs.

### Nonunion - Treatment

The “gold standard” or preferred option in nonunion treatment is the removal of necrotic bone tissue and stabilization with internal or external fixation devices, in most cases, accompanied by bone grafts to stimulate osteogenesis in indolent bone ends.^30^ However, union is not always accomplished after a surgical treatment, with reported success rates varying from 68% to 96% for the first surgical procedure, depending on the fracture location and surgical method.^31^–^35^

Currently, several nonoperative methods have been developed for treatment of nonunions that avoid the risks associated with surgery. These alternative treatments include LIPUS, electrical stimulation, and extracorporeal shock-wave therapy. Success rates of electrical stimulation techniques on nonunions do not seem to differ greatly from those of surgical procedures, with reported rates ranging from 65% to more than 80%.^36^–^40^ In addition, equivalent success rates (52–91%) have been reported on extracorporeal shock-wave therapy.^41^–^44^ Despite their good results and the fact that most surgeons believe that therapeutic ultrasound may assist in fracture healing, current usage of alternative treatments is rare, primarily because of the perceived lack of evidence and availability.^45^

#### Mechanisms of LIPUS on fracture healing

Ultrasound, a form of mechanical energy that can be transmitted in organisms as high-frequency acoustical pressure waves, has been widely used as a therapeutic, operative, and diagnostic tool.^46^,^47^ Used with an intensity of 1-3 W/cm^2^, ultrasound can cause considerable heat in tissues and is therefore used to decrease joint stiffness and improve muscle mobility.^48^ For diagnostic or imaging purposes, even lower ultrasound intensities (1-50 mW/cm^2^) are used, which are considered nonthermal and nondestructive.^49^ Although the absorption of the energy from LIPUS causes very little (<1°C) heating, some enzymes, such as matrix metalloproteinase (MMP) -1, or collagenase, are sensitive to this variation in temperature.^49^,^50^ Therefore, ultrasound may alter enzymatic processes associated with fracture healing. In addition, an increased blood flow to dissipate the accumulated heat and return the tissue to homeostatic levels has been attributed to the small increase in temperature.^51^

Next to thermal effects, LIPUS induced bone osteogenesis may also be ascribed to nonthermal processes, including acoustic streaming and cavitation. Cavitation “involves the pulsation of gas or vapor-filled voids in a sound field”^52^ with the accumulation of gas and formation of gas bubbles.^53^ At the surface of such a gas bubble, acoustic streaming can occur,^54^ which has been found to affect diffusion rates and membrane permeability.^55^

Duarte was the first to develop the LIPUS (intensity 30 mW/cm^2^) for fracture repair and demonstrate in an experimental animal study with 45 rabbits that this therapy caused a significant increase of callus.^3^ Later, Pilla *et al*. demonstrated that LIPUS (20 minutes daily, 200 μs burst of 1.5 MHz sine waves repeated at 1 kHz) with an intensity of 30mW/ cm^2^ not only had an effect on the quantity of callus, but also significantly increases the mechanical strength and stiffness of the callus.^4^

Although it is still not completely understood by which specific mechanism LIPUS accelerates and enhances the fracture repair process, there are indications that it has influence on the different stages of the healing process, including signal transduction, gene expression, and blood flow.^5^ As suggested by several *in vivo* experimental studies, LIPUS rather affects the earlier inflammation or callus formation stage than the later occurring remodeling phase.^5^,^56^ The increased quantity of callus after LIPUS treatment has been shown by histological analysis to be due to increased endochondral bone formation processes.^57^ Pilla *et al.*^4^ found that torque and torsional stiffness of osteotomies were significantly greater in the LIPUS treated group compared with the untreated group in the first three weeks after surgery, but there was no difference between the groups at 4 weeks follow-up. This confirms the hypothesis that LIPUS treatment only stimulates the earlier phases of fracture healing. Also in another animal osteotomy model, LIPUS treatment was found to increase bone mineral density at 75 days after surgery, but this effect was not seen at 120 days after surgery.^56^

In vitro studies have shown that ultrasound can induce conformational changes in the cell membrane, resulting in changes in ionic permeability^58^,^59^ and second messenger activity.^60^,^61^ However, if the ultrasound signal influences the rate of healing, it must also show an effect on the expression of specific genes involved in the healing process. Using differential mRNA display on callus material from a rat bilateral femur fracture,^5^ it was shown that several genes were upregulated during exposure to ultrasound. These include genes encoding for the matrix protein osteopontin and a growth factor inducible gene that plays a role during chondrogenesis.

Additionally, ultrasound has been shown to increase angiogenesis. In vitro cell studies have demonstrated that LIPUS exposure leads to an increased expression of vascular endothelial growth factor-A levels in osteoblasts.^62^ Also, LIPUS has been shown to increase the production of fibroblast growth factor and interleukin-8 in osteoblasts and periostal cells.^63^,^64^ These cytokines stimulate and are necessary for angiogenesis, which is a key component in the earliest stages of bone repair. The role of LIPUS in angiogenesis is supported by the observation that factors diminishing blood flow (smoking, vascular problems, diabetes) have a negative influence on fracture healing.

In the fracture repair process, proliferation of osteoblasts and chondrocytes serves as a trigger for the following sequential cellular events.^65^ Takayama *et al*. found a substantial effect of LIPUS on the differentiation of rat osteosarcoma cells, but no effect on the proliferation of these cells.^66^ Therefore, it seems unlikely that LIPUS can initiate the fracture repair process, which might be the reason that LIPUS has yet been used more on fresh fracture than on nonunions.^17^

### Pre-clinical studies

Multiple animal studies have been conducted to examine the effect of LIPUS on fracture repair. These studies include the investigation of the macroscopic effect of LIPUS on fracture models, but also research at the extracellular and intracellular level. In most animal nonunion models, segmental bone resection has been used to induce a nonunion. Takikawa *et al*. evaluated the effect of LIPUS on rat tibial nonunions by placing a portion of the tibialis muscle within the fracture site, preventing the two ends of the bone from bridging.^67^ Using this technique, the nonunion was believed to more accurately mimic a clinical nonunion. Results of this study showed that 50% of LIPUS-treated nonunions healed after 6 weeks, compared with none of the tibial fractures in the control group.

Using a bilateral closed femoral fracture model in rats, Azuma *et al*. tried to determine the influence of LIPUS at different stages of fracture healing.^68^ They found that union was accelerated regardless of the duration or timing of the treatment. This animal experiment suggests that no specific stage of fracture healing is more sensitive than another, and therefore that LIPUS might be useful in every stage of fracture repair.

Animal studies are commonly used as an intermediate between cell studies and clinical studies. For example, the increased angiogenesis findings in cell studies are supported by Rawool *et al.*, who used midshaft ulnar fractures in dogs and found a threefold increase in blood flow after LIPUS treatment for one week of 20 minutes daily.^69^

### Clinical studies

With the first clinical application of their developed LIPUS signal, Xavier and Duarte obtained a healing rate of 70% in 26 nonunions.^70^ Since then, most studies have been performed to study the effect of LIPUS on healing of fresh fractures, many in a randomized controlled fashion. The first double-blind RCT of LIPUS on fresh tibial fractures was performed by Heckman *et al*., who's results showed a significant (38%) reduction in time to healing in the LIPUS treatment group compared to the control group.^71^ Several other studies have reported similar favorable results.^8^,^9^ Busse *et al*. conducted a meta-analysis of the available RCTs on the effect of LIPUS on healing time of fresh fractures.^10^ Review of the pooled data from the three studies included in the analysis revealed a difference of 64 days between the LIPUS treatment and control groups.

Almost no RCTs have been conducted to study the effect of LIPUS on healing of nonunions, since it is generally considered unethical to conduct placebo-controlled studies on their treatment. Namely, this would mean denying a patient a treatment of his nonunion for another six to nine months.^18^ Therefore, most studies on treatment of nonunions use self-paired controlled designs. These designs are valid and medically appropriate to study treatment effects in medical conditions with unfavorable prognoses, such as nonunions, which fail to heal without any further treatment.^16^ The effectiveness of the LIPUS treatment can be estimated by the healed status change, where the patient serves as his or her own control.^72^ In addition, self-pairing offers the advantage of minimizing sources of variability between the treatment and control groups, which minimizes confounding bias.^18^,^20^

Despite the lack of evidence from RCTs, the use of LIPUS for the treatment of established nonunions has been approved by the FDA in February 2000,^12^ based on the results of three prospective self-paired studies.^16^,^20^,^73^ The average healing rate of these studies was 81.2% among nonunions with a mean fracture age of 21.3 months.

### Healing rate and time to healing

In our review, we found an average healing rate of 87% of nonunions among eight studies, which is comparable to the 85% healing rate among 700 nonunions reported in a registry of all orthopedic prescription use of LIPUS since FDA approval of Exogen's Sonic Accelerated Fracture Healing System (SAFHS) (Exogen, Inc, Piscataway, NJ) for marketing.^22^ In addition, Rubin *et al*. reported a similar healing rate in their own review of the prescription use registry as of June 2000.^74^ The 1546 nonunions had a healing rate of 83%, with an average time to healing of 172 days, which is comparable to the mean healing time of the included studies from our review (146.5 days). Although the RCT from our review reported a 100% healing rate for both the active and placebo group, a significant difference in time to healing was demonstrated, favoring the active LIPUS treatment group.^15^

### Factors influencing healing rate

The largest study reviewed by us^20^ contained 366 nonunions of various bones and found a healing rate of 86%. Because various bones were investigated and the sample size was relatively large, separate healing rates for the different locations of nonunions could be calculated, ranging from 69% for humeral nonunions to 100% for scaphoid nonunions. This is consistent with the results of Ricardo *et al*.,^15^ who also found a 100% healing rate in 21 scaphoid nonunions. Scaphoid nonunions do not only seem to heal more often, but also in a shorter time, as Pigozzi *et al*. demonstrated a significant shorter healing time in scaphoid nonunions than in the nonunions located elsewhere.^21^

In the study of Mayr *et al*.^20^ it was observed that the success rate consistently decreased from 97% among 20-year-old patients to 71% among 70-year-old patients. Further, they found a variation in healing rates depending on the use of described drugs. For example, in patients treated with calcium channel blockers, the nonunions healed in only 63%.

With respect to the effect of smoking on the healing rate of nonunions, different studies show conflicting results. Mayr *et al*.^20^ found a healing rate of 79% among active smokers, compared with 89% among those who stopped smoking and 87% who never smoked. However, the comparison of smoking strata in the study of Nolte *et al*.^16^ was significant (*P* < 0.05), with healing rates of 60%, 82% and 100% among active smokers, those who stopped smoking, and nonsmokers, respectively.

Jingushi *et al*.^17^ recommend in their article to start with LIPUS treatment within 6 months of the most recent operation. This recommendation is based on their findings of a significant higher healing rate for those with a shorter time period between the most recent operation and start of LIPUS treatment, with rates decreasing from 89.7% for a time period of three to six months to 52.6% for ≥12 months. However, Gebauer *et al*.^18^ did not find a difference in healing rate between nonunions with a last procedure interval of 120–365 days and 366–730 days (88% and 100%, respectively).

### Advantages and disadvantages of LIPUS

The primary advantage of LIPUS is the fact that it is a safe, noninvasive treatment which prevents patients from surgical risks.^6^,^7^ However, this also accounts for other alternative treatments of fracture healing, including extracorporeal shock wave therapy and electrical stimulation. Although similar healing rates have been found for all three (LIPUS, shock wave therapy,^41^–^44^ and electrical stimulation^36^–^40^) alternative methods, LIPUS has some advantages over the other two treatment options, i.e., no hospital admission is required for LIPUS, which is in contrast with shock wave therapy.^6^ Compliance rates are high, with a daily treatment session duration of only 20 minutes, compared with many hours for electrical or electromagnetic stimulation.^75^

Although LIPUS treatment is relatively expensive, total costs are still lower than those associated with surgical interventions, with an estimated overall cost saving of US$13,000–15,000 per patient.^76^

Despite the fact that the need for additional surgery is eliminated, nonunions treated with LIPUS still require an average of five months to heal [Table 1]. However, considering that established nonunions already failed to heal for approximately 9 months, this time is reasonable, also because daily activities can be continued during the time of treatment.

### Study limitations

There are some limitations in this review. First, only one randomized controlled double-blinded trial was included. This RCT had a small sample size and included only scaphoid nonunions, which limits the generalizability of its results. Most included trials were self-paired case series without blinding, raising the potential for measurement bias. However, confounding bias was minimized, as the patients served as their own control. The disadvantage of a self-paired design is, however, that it is not possible to compare LIPUS with either no further treatment or with other treatments in the same or comparable patients. To assess the comparative efficacy of LIPUS nonunions, LIPUS treatment should rather be compared with a surgical treatment, such as internal fixation.^77^ This study design would be ethically acceptable, since surgery is the current gold standard for the treatment of nonunions.

Second, the average healing rate reported in this review might not be valid, because the studies are heterogeneous regarding nonunion location and patient population. On the other hand, this healing rate might be generalizable to a large population of nonunions, as studies on various bones were included in this review.

Also, the included studies mostly contained a small or very small sample size, except for one.^20^ Therefore, it is questionable to what extent their separate results can be used in deciding whether to use LIPUS as a treatment for nonunions. Thus, it would be desirable to pool data from these small sample sized studies. However, since there is little homogeneity among the current published trials on LIPUS treatment of nonunions, no meta-analysis has been performed in this area yet.

## CONCLUSION

With an average healing rate of approximately 87%, LIPUS appears to be a safe and cost-effective alternative to surgery for the treatment of nonunions. Although the exact mechanism of LIPUS on fracture healing is still unknown, several biological and physical changes have been recognized. Several factors have been demonstrated that may impair the effect of LIPUS on fracture healing. However, still substantially high healing rates have been observed when impairing factors were present. The main advantage of LIPUS is the avoidance of surgical risks and morbidity, without a decrease in healing rate. Although similar success rates have been demonstrated for other alternative fracture healing treatments, LIPUS has the additional advantages over electrical stimulation and extracorporeal shock wave therapy of shorter daily treatment duration, and the application on an outpatient basis, respectively. Unfortunately, almost no RCTs or large case series are performed on this issue, and pooling of the available small-sized case series is not valid due to heterogeneity among the studies. Therefore, more homogeneous and larger controlled series or RCTs are needed to investigate the efficacy of LIPUS in nonunions.
